# Exploring the physical layer frontiers of cellular uplink: The Vienna LTE-A Uplink Simulator

**DOI:** 10.1186/s13638-016-0609-1

**Published:** 2016-04-27

**Authors:** Erich Zöchmann, Stefan Schwarz, Stefan Pratschner, Lukas Nagel, Martin Lerch, Markus Rupp

**Affiliations:** 1 Institute of Telecommunications, TU Wien, Vienna, 1040 Austria; 2 Christian Doppler Laboratory for Dependable Wireless Connectivity for the Society in Motion, Vienna, Austria

**Keywords:** 4G mobile communication, Cellular uplink, Computer simulation

## Abstract

Communication systems in practice are subject to many technical/technological constraints and restrictions. Multiple input, multiple output (MIMO) processing in current wireless communications, as an example, mostly employs codebook-based pre-coding to save computational complexity at the transmitters and receivers. In such cases, closed form expressions for capacity or bit-error probability are often unattainable; effects of realistic signal processing algorithms on the performance of practical communication systems rather have to be studied in simulation environments. The Vienna LTE-A Uplink Simulator is a 3GPP LTE-A standard compliant MATLAB-based link level simulator that is publicly available under an academic use license, facilitating reproducible evaluations of signal processing algorithms and transceiver designs in wireless communications. This paper reviews research results that have been obtained by means of the Vienna LTE-A Uplink Simulator, highlights the effects of single-carrier frequency-division multiplexing (as the distinguishing feature to LTE-A downlink), extends known link adaptation concepts to uplink transmission, shows the implications of the uplink pilot pattern for gathering channel state information at the receiver and completes with possible future research directions.

## Introduction

Current cellular wireless communications employs Universal Mobile Telecommunications System (UMTS) Long Term Evolution (LTE) as the high data rate standard [1]. The increasing demand of high data traffic in up- and downlink forces engineers to push the limits of LTE [2], e.g. through enhanced multi-user multiple input, multiple output (MIMO) support [3, 4], coordinated multipoint (CoMP) transmission/reception [5, 6] as well as improved channel state information (CSI) feedback algorithms [7]. The authors of [8] predict further evolution of existing LTE/LTE-Advanced (LTE-A) systems in parallel to the development of new radio-access technologies operating at millimetre wave frequencies even beyond the expected roll-out of 5G technologies by 2020. Fair comparison of novel signal processing algorithms and transceiver designs has to assure equal testing and evaluation conditions to enable reproducibility of results by independent groups of researchers and engineers [9]. For performing system-level simulations, [10, 11] or [12] are freely accessible options. For link level, multiple commercial products are available that facilitate reproducible research, such as, *is-wireless* LTE PHY LAB [13] or *Mathworks* LTE System Toolbox [14] and some non-commercial projects which were introduced in [15] and [16]. To the best of the authors’ knowledge, however, the Vienna LTE simulators are the only MATLAB-based suite of simulation tools including LTE system and link level, publicly available under an academic use licence, thus, free of charge for academic researchers all over the world. The software suite consists of three simulators. The downlink link and system level simulators are comprehensively studied in [2, 9, 17]. In this paper, we introduce the latest member of the family of Vienna LTE Simulators, that is, the Vienna LTE-A Uplink Link Level Simulator, downloadable at [18], and highlight our research conducted by means of this simulator.

### Outline and contributions

We start with a brief re-capitulation of the LTE-A specifics and introduce the modulation and multiple access scheme and the employed MIMO signal processing of LTE-A uplink in Section 2. We then develop a matrix model describing the input-output relationship of the LTE-A uplink and present signal-tointerference-and-noise ratio (SINR) expressions for single-carrier frequency-division multiplexing (SC-FDM) as well as orthogonal frequency-division multiplexing (OFDM). The OFDM SINR expression and the performance of OFDM will serve as reference to study the effects of discrete Fourier transform (DFT) spreading imposed by SC-FDM.

In Section 3, we investigate the physical layer performance of SC-FDM and OFDM, comparing bit error ratio (BER) and peak-to-average power ratio (PAPR). The BER for LTE single input single output (SISO) transmissions was already analysed in link-level simulations by [19–21] and semi-analytically by [22, 23]. By means of our simulator, we reproduce these results and provide bounds to predict the performance of SC-FDM with respect to OFDM. The insights gathered by the BER simulations allow us to interpret the difference in throughput obtained by OFDM and SC-FDM, as discussed in Section 4.

Based on the SINR expressions developed in Section 2, we present a limited feedback strategy for link adaptation in Section 4 and contrast the performance of LTE uplink with channel capacity and other performance upper bounds that account for practical design restrictions [24]. Until Section 5, we assume perfect CSI at the receiver. The remaining sections will describe methods to obtain CSI at the receiver.

In Section 5, we highlight and describe the demodulation reference signal (DMRS) structure employed in LTE-A uplink to facilitate channel estimation of the time-frequency selective wireless channel.

Based on the obtained insights, we elaborate on the basic concept of DFT-based time domain channel estimation in Section 6 and review alternative code/frequency domain methods that can outperform DFT-based schemes [25].

Due to the increasing number of mobile users that stay connected while travelling in cars or (high speed) trains, we then shift our focus to high velocity scenarios. Such scenarios entail high temporal selectivity of the wireless channel, rendering accurate channel interpolation very important to sustain reasonable quality of service. We introduce and investigate basic concepts of channel interpolation in Section 7.

We briefly discuss open questions for future research in Section 8 and conclude in Section 9. Details to the handling of the simulator are provided in [26].

### Notation

Matrices are denoted by bold uppercase letters such as ***H*** and vectors by bold lowercase letters such as ***h***. The entries of vectors and matrices are accessed by brackets and subscripts, e.g. [***h***]_*k*_ and [***H***]_*k*,*n*_. Spatial layers or receive antennas are denoted by superscripts in braces, e.g. ***x***^(*l*)^. The superscripts (·)^*T*^ and (·)^*H*^ express transposition and conjugate transposition. ∥·∥_2_, $\| \cdot \|_{\infty }$ and ∥·∥_*F*_ symbolize the Euclidean norm, the Maximum norm and the Frobenius norm, respectively. The entrywise (Hadamard) product is denoted by ⊙ and the Kronecker product by ⊗. The all ones vector/matrix is denoted by . The operator ***X***=Diag(***x***) places the vector ***x*** on the main diagonal of ***X***, and conversely, the operator ***x***=diag(***X***) returns the vector ***x*** from the main diagonal of ***X***. A block-wise Toeplitz (circulant, diagonal) matrix is a block matrix with each matrix of Toeplitz (circulant, diagonal) shape. The size of matrices is expressed via their subscripts, whenever necessary.

## LTE-specific system model and SINR

(1) $$ {\fontsize{9.2}{6} \begin{aligned} \boldsymbol{\hat{x}} &= \left(\boldsymbol{I}_{L} \otimes \boldsymbol{D}_{N_{SC}}^{H} \right) \boldsymbol{F} \left(\boldsymbol{I}_{N_{R}} \otimes \boldsymbol{M}^{H} \boldsymbol{D}_{N_{\text{FFT}}} \boldsymbol{P}_{\text{remCP}}\right)\\ &\quad\times\boldsymbol{H} \left(\boldsymbol{I}_{N_{T}} \otimes \boldsymbol{P}_{\text{addCP}}\boldsymbol{D}_{N_{\text{FFT}}}^{H} \boldsymbol{M}\right)\left(\boldsymbol{W} \otimes \boldsymbol{I}_{N_{\text{SC}}} \right)\left(\boldsymbol{I}_{L} \otimes \boldsymbol{D}_{N_{\text{SC}}} \right)\boldsymbol{x}\\ &\quad+ \underbrace{\left(\boldsymbol{I}_{L} \otimes \boldsymbol{D}_{N_{\text{SC}}}^{H} \right) \boldsymbol{F}\left(\boldsymbol{I}_{N_{R}} \otimes \boldsymbol{M}^{H} \boldsymbol{D}_{N_{\text{FFT}}} \boldsymbol{P}_{\text{remCP}}\right) \boldsymbol{n}}_{\tilde{\boldsymbol{n}}} \\ &= \overbrace{\left(\boldsymbol{I}_{L} \otimes \boldsymbol{D}_{N_{\text{SC}}}^{H} \right)}^{\substack{\boldsymbol{I}_{L\,N_{\text{SC}}} \\ \text{for OFDM}}} { \boldsymbol{F} \boldsymbol{H}_{\text{eff}}} \overbrace{\left(\boldsymbol{I}_{L} \otimes \boldsymbol{D}_{N_{\text{SC}}} \right)}^{\substack{\boldsymbol{I}_{L\,N_{\text{SC}}} \\ \text{for OFDM}}} \boldsymbol{x} + \tilde{\boldsymbol{n}} \\ &= \boldsymbol{K} \boldsymbol{x} + \tilde{\boldsymbol{n}} = \quad \underbrace{\boldsymbol{I}\odot \boldsymbol{K} \boldsymbol{x}}_{\text{desired signal}} \quad + \quad \underbrace{\left(\boldsymbol{K}-\boldsymbol{I}\odot \boldsymbol{K}\right) \boldsymbol{x}}_{\text{intra- and interlayer interference}} \\&\quad+ \tilde{\boldsymbol{n}}~. \end{aligned}}  $$

**Fig. 1:**
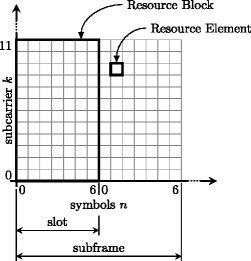
The LTE-A uplink resource grid

LTE operates on a time-frequency grid as shown in Fig. 1. The number of subcarriers is always a multiple of 12; 12 adjacent subcarriers over 7(or 6—in case of extended CP) successive OFDM symbols are called resource block (RB). Each RB thus consists of 12×7 (12×6) resource elements (REs), corresponding to the different time-frequency bins. A detailed description of LTE up- and downlink is available, e.g. in [27].

**Fig. 2:**
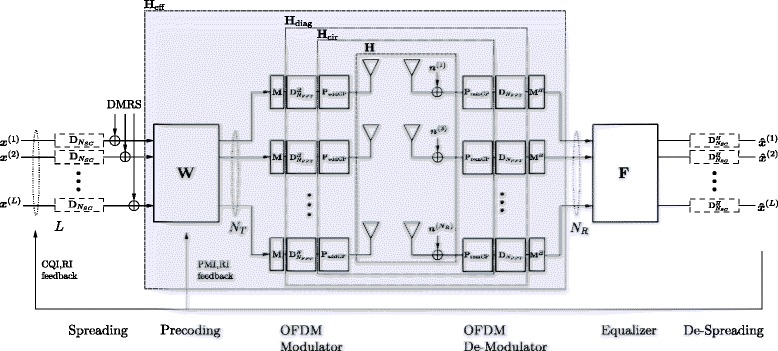
The LTE-A uplink transceiver

We focus on those details necessary to describe our system model at time *n*^1^. LTE employs OFDM(A)^2^ as physical layer modulation and multiple access scheme in the downlink and SC-FDM(A), i.e. DFT-spreaded OFDM, in the uplink. In a SC-FDM model, OFDM can be considered a special case. The major difference is an additional spreading and de-spreading stage at the transmitter and receiver, highlighted via dashed boxes in Fig. 2. The common parts of the system model will be described from left to right.

Right after the DFT spreading, the DMRS is inserted. The DMRS will be considered later for the purpose of channel estimation (CE). Next, MIMO precoding is carried out, exploiting a set of semi-unitary precoding matrices ***W***, pooled in the precoder codebook $\mathcal {W}$, as defined in [1]. For LTE-A uplink transmission, the precoding matrix applied for a given user is equal for all RBs assigned to this user. In case of spatial multiplexing, each spatial layer is transmitted with equal power.

Each antenna is equipped with its own OFDM modulator, consisting of subcarrier mapping, inverse fast Fourier transform (IFFT) and a CP addition. To cope with the channel dispersion and to avoid Intersymbol Interference (ISI), LTE employs a CP. As a result of multipath propagation, a previous symbol may overlap with the present symbol, introducing ISI and impairing the orthogonality between subcarriers, i.e. causing Intercarrier Interference (ICI) [28]. Normal and extended CP lengths, with a respective duration of 4.7 and 16.7 *μ*s, are standardized, enabling a simple trade-off between ISI immunity and CP overhead.

At the transmitter, the processing occurs in a reversed order. First, the OFDM demodulation/FFT takes place to get back into the frequency domain. The immunity to multipath propagation (stemming from the CP) allows to employ one-tap frequency domain equalizers ***F*** without performance loss. At last, de-spreading delivers the data estimates.

All this previously informally described processing is linear, and we are able to formulate a matrix-vector input-output relationship between a (stacked) data-vector ***x*** and its estimate $\boldsymbol {\hat {x}}$. For simplicity, we assume that the channel stays constant during one OFDM symbol. A detailed system description based on [29] can be found in [30].

In order to adapt the data transmission to the current channel state, LTE-A applies limited feedback; a comprehensive specification follows in Section 4. Limited feedback is depicted via the feedback arrow in Fig. 2. The data vector $\boldsymbol {x}^{(l)} \in \mathbb {C}^{N_{\text {SC}}\times 1}$ of layer *l*∈{1,…,*L*} contains modulated symbols for each of the *N*_SC_ subcarriers. The number of transmit layers depends on the LTE-A specific rank indicator (RI) feedback. The data symbols are coded with a punctured turbo code whose rate is determined by the channel quality indicator (CQI). Subsequently, the codewords are mapped onto a quadrature amplitude modulation (QAM) alphabet (4/16/64 QAM), where the size of the alphabet depends on the CQI as well. All ***x***^(*l*)^ are stacked into one vector $\boldsymbol {x}\in \mathbb {C}^{N_{\text {SC}}L\times 1}$ on which layer-wise spreading and joint precoding—according to the precoding matrix indicator (PMI)—of all subcarriers take place. The subsequent OFDM modulator consists of the localized subcarrier mapping ***M***, mapping *N*_SC_ subcarriers to the centre of an *N*_FFT_ point IFFT and the addition of the CP.

**Fig. 3:**
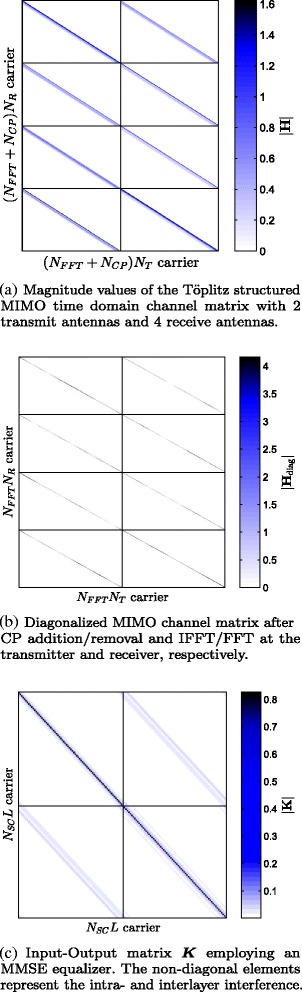
Examples of different channel abstractions

Depending on the level of abstraction, our system model can be described via different channel matrices. The physical baseband time domain channel is described by a block-wise Töplitz matrix $\boldsymbol {H} \in \mathbb {C}^{(N_{\text {FFT}}+N_{\text {CP}})N_{\mathrm {R}} \times (N_{\text {FFT}}+N_{\text {CP}})N_{\mathrm {T}}}$, with *N*_T_ transmit and *N*_R_ receive antennas, which turns block-wise circulant (***H***_cir_) after addition (***P***_addCP_) and removal (***P***_remCP_) of an appropriately chosen CP of length *N*_CP_. Finally, it turns diagonal after the IFFT and FFT on the transmitter and receiver, respectively. An example of the Töplitz and diagonal structured channel is demonstrated in Fig. 3a, b, respectively.

(2) $$ \boldsymbol{H}_{\text{diag}} = \left(\boldsymbol{I}_{N_{R}} \otimes \boldsymbol{D}_{N_{\text{FFT}}} \boldsymbol{P}_{\text{remCP}}\right) \boldsymbol{H} \left(\boldsymbol{I}_{N_{T}} \otimes \boldsymbol{P}_{\text{addCP}}\boldsymbol{D}_{N_{\text{FFT}}}^{H} \right)  $$

The last step of the OFDM de-modulator is the reversal of the localized subcarrier mapping ***M***^*H*^. The effective MIMO channel from *L* transmit layers to *N*_R_ receive antennas, incorporating the precoder, the OFDM modulator, the time-domain MIMO channel ***H*** and the OFDM de-modulator, is abstracted to one block matrix ***H***_eff_. This greatly facilitates the readability of all formulas later on.

(3) $$ \boldsymbol{H}_{\text{eff}} = \left(\boldsymbol{I}_{N_{R}} \otimes \boldsymbol{M}^{H} \right) \boldsymbol{H}_{\text{diag}} \left(\boldsymbol{I}_{N_{T}} \otimes \boldsymbol{M}\right) \left(\boldsymbol{W} \otimes \boldsymbol{I}_{N_{\text{SC}}} \right)   $$

The additive noise is assumed independent across antennas and is distributed zero mean, white Gaussian $\boldsymbol {n}^{(i)} \sim \mathcal {CN}\lbrace \boldsymbol {0}, {\sigma _{n}^{2}} \boldsymbol {I} \rbrace, \;\; i \in \lbrace 1,\dots, N_{\mathrm {R}}\rbrace $. The stacked noise vector $\boldsymbol {n}=\left (\left (\boldsymbol {n}^{(1)}\right)^{T}, \dots, \left (\boldsymbol {n}^{(N_{\mathrm {R}})}\right)^{T} \right)^{T}$ is thus zero mean, white Gaussian as well.

The frequency domain one-tap equalizer^3^***F*** is chosen conforming to different criteria, either the zero forcing (ZF) criterion, which removes all channel distortions at risk of noise enhancement, or the minimum mean squared error (MMSE) criterion, which tries to minimize the effects of noise enhancement and channel distortion.

After the de-spreading operation, the data estimates $\boldsymbol {\hat {x}}$ of the noisy, received signal are given in Eq. (1), with the beforementioned convenient abbreviation (3), and $\boldsymbol {D}_{N_{\text {FFT}}}$ is the DFT matrix of size *N*_FFT_.

### SC-FDM SINR

The special structure of Eq. (1), due to the frequency domain one-tap equalizer and the DFT spreading, yields a block-wise circulant input-output matrix, cf. Fig. 3c,

(4) $$ \boldsymbol{K} = \left(\boldsymbol{I}_{L} \otimes \boldsymbol{D}_{N_{\text{SC}}}^{H} \right) \boldsymbol{F} \boldsymbol{H}_{\text{eff}} \left(\boldsymbol{I}_{L} \otimes \boldsymbol{D}_{N_{\text{SC}}} \right)~.  $$

This block-wise circulant structure produces a constant post equalization and post spreading SINR over all subcarriers within one layer [30]. The detailed derivation is provided in the Appendix. 
(5)

where

(6) $$  \boldsymbol{S}^{(l)}=\left(\boldsymbol{0} \quad \boldsymbol{I}_{N_{\text{SC}}} \quad \boldsymbol{0} \right)~,  $$

selects that part of ***FH***_eff_ effecting the *l*th layer. The second moment (power) of the zero mean transmit symbols is depicted by ${\sigma _{x}^{2}}$.

### OFDM SINR

In contrast to SC-FDM, no spreading takes place for OFDM. The dashed boxes in Fig. 2 are replaced by identity matrices; they are simply omitted. Thus, different subcarriers *k* are orthogonal/independent and the equalizer treats the corresponding subcarrier channel ***H***_*k*_ only. We use the subscript *k* to denote the relevant part of the full channel matrix ***H***_eff_ for the *k*th subcarrier. The corresponding indices within the diagonal matrix ***H***_diag_ are , with the canonical base vectors ***e***_*k*_. Using this notation, the effective subcarrier channel $\boldsymbol {H}_{k} \in \mathbb {C}^{N_{R}\times L}$ is 
(7)

and ***F***_*k*_ is its linear one-tap equalizer. The SINR formula is quite similar to the SC-FDM case, except that the SINR shows subcarrier dependency now. The SINR vector at layer *l* reads

(8) $$\begin{array}{@{}rcl@{}} &\left[\boldsymbol{\operatorname{\mathbf{SINR}}}^{\text{OFDM},\;{(l)}}\right]_{k}=  \\ &\frac{{\sigma_{x}^{2}} \big|\boldsymbol{s}^{(l)}\text{diag} \left(\boldsymbol{F}_{k} \boldsymbol{H}_{k} \right)\! \big|^{2} }{ { {\sigma_{x}^{2}} \| \boldsymbol{s}^{(l)} \boldsymbol{F}_{k}\boldsymbol{H}_{k} \|_{2}^{2}-{\sigma_{x}^{2}} \big|\boldsymbol{s}^{(l)} \text{diag} \left(\boldsymbol{F}_{k} \boldsymbol{H}_{k} \right)\!\big|^{2} }+{\sigma^{2}_{n}} \| \boldsymbol{s}^{(l)} \boldsymbol{F}_{k} \|_{2}^{2}} ~, \notag \end{array} $$

with the selection vector

(9) $$ \boldsymbol{s}^{(l)} = \left(\!\begin{array}{ccc} {0} \dots 0 & {1} & 0 \dots {0} \end{array}\! \right) ~,  $$

with appropriate number of zeros and a one at the *l*th position.

## SC-FDM features

We first discuss the main reason to apply SC-FDM at uplink transmissions, namely PAPR. Then, we look at the expenses of employing it. We will see a worse performance of the coded transmission.

### Peak-to-average-power ratio

SC-FDM is employed as the physical layer modulation scheme for LTE uplink transmission, due to its lower PAPR compared to OFDM [31]. Lower PAPR, or similarly lower crest factor, leads to reduced linearity requirements for the power amplifiers and to relaxed resolution specifications for the digital-to-analogue converters at the user equipments, entailing higher power efficiency.

The Vienna LTE-A uplink simulator calculates the discrete-time baseband PAPR with the default oversampling factor *J*=4 [32]. The discrete time signal on transmit antenna $t \in \lbrace 1,\dots,N_{\mathrm {T}}\rbrace $ is therefore calculated as

(10) $$\begin{array}{@{}rcl@{}} \left[\boldsymbol{s}_{\text{tx}}^{(t)}\right]_{m}=\frac{1}{\sqrt{N_{_{\text{FFT}}}}} \sum_{k=0}^{N_{\text{FFT}}-1} \!\!\!\!\left[\boldsymbol{x}_{\text{pre}}^{(t)}\right]_{k} e^{j\frac{2\pi m k}{J N_{\text{FFT}}}}, \\ \;\; 0 \le m \le J N_{_{\text{FFT}}}-1 ~, \notag \end{array} $$

where $\boldsymbol {x}_{\text {pre}}^{(t)}$ is the transmit vector right after precoding and before the IFFT at transmit antenna *t*. The PAPR of the stacked vector $\boldsymbol {s}_{\text {tx}}=\left (\left (\boldsymbol {s}_{\text {tx}}^{(1)}\right)^{T}, \dots, \left (\boldsymbol {s}_{\text {tx}}^{(N_{\mathrm {T}})}\right)^{T} \right)^{T}$ is calculated as

(11) $$\begin{array}{@{}rcl@{}} \operatorname{PAPR}\{\boldsymbol{s}_{\text{tx}}\}&= \frac{\max\limits_{1\le t \le N_{\mathrm{T}}} \max\limits_{0 \le m \le J N_{_{\text{FFT}}}-1}\left(\big|\big[\boldsymbol{s}_{\text{tx}}^{(t)}\big]_{m} \big|^{2} \right)}{\mathbb{E}_{t}\left\{\mathbb{E}_{n}\big\lbrace\big|\big[\boldsymbol{s}_{\text{tx}}^{(t)}\big]_{m} \big|^{2} \big\rbrace\right\}} \\ &\approx N_{\mathrm{T}} N_{_{\text{FFT}}} \| \text{diag}\left(\boldsymbol{s}_{\text{tx}} \boldsymbol{s}_{\text{tx}}^{H} \right)\|_{\infty} \big/ \|\boldsymbol{s}_{\text{tx}} \|_{2}^{2}~,\notag \end{array} $$

where the Euclidean norm in the denominator serves as an estimate for the ensemble average.

**Fig. 4:**
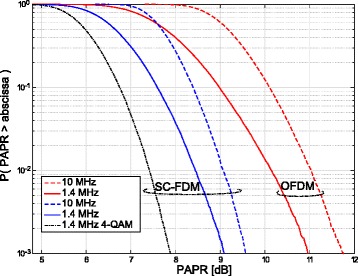
PAPR for SC-FDM and OFDM for different bandwidths (1.4 and 10 MHz) and modulation alphabets (4/64 QAM)

Figure 4 depicts the PAPR of OFDM and SC-FDM obtained for different system bandwidths. Already for a small bandwidth (1.4 MHz), there is a significant reduction for SC-FDM over OFDM. With increasing bandwidth, OFDM’s PAPR grows and the gains obtained by SC-FDM become more and more pronounced. The PAPR also depends on the modulation alphabet; the smaller the alphabet, the smaller the PAPR. This effect is illustrated in dotted lines in Fig. 4, where we have shown the PAPR of 4-QAM, exemplarily.

### BER comparison over frequency selective channels

The additional spreading of SC-FDM leads to an SINR expression that is constant on all subcarriers as for single-carrier transmission, legitimating its name. The aim of this subsection is to analyse the SINR expression more carefully for the SISO case^4^ and draw conclusions on BER performance.

We focus on the two most prominent equalizer concepts and start with the ZF equalizer, for whom the SC-FDM signal-to-noise ratio (SNR) expression (??) reduces to the harmonic mean

(12) $$ {\operatorname{SNR}}_{\text{ZF}}^{\text{SC-FDM}}=\frac{{\sigma_{x}^{2}}}{{\sigma^{2}_{n}}} \frac{1}{\frac{1}{N_{\text{SC}}}\sum\limits_{k=1}^{N_{\text{SC}}} \frac{1}{|\boldsymbol{H}_{k}|^{2}}} ~,   $$

whereas the OFDM expression (8) is sub-carrier dependent and becomes proportional to the channel transfer function

(13) $$ \left[\boldsymbol{\operatorname{SNR}}_{\text{ZF}}^{\text{OFDM}}\right]_{k}=\frac{{\sigma_{x}^{2}}}{{\sigma^{2}_{n}}} |\boldsymbol{H}_{k}|^{2} ~.  $$

The average OFDM SNR

(14) $$  \overline{{\operatorname{SNR}}_{\text{ZF}}^{\text{OFDM}}}=\frac{{\sigma_{x}^{2}}}{{\sigma^{2}_{n}}} \frac{1}{N_{\text{SC}}}\sum\limits_{k=1}^{N_{\text{SC}}} |\boldsymbol{H}_{k}|^{2}  $$

yields an upper bound on the SC-FDMA SNR due to the harmonic mean—arithmetic mean inequality [33].

(15) $$  {\operatorname{SNR}}_{\text{ZF}}^{\text{SC-FDM}} \le \overline{{\operatorname{SNR}}_{\text{ZF}}^{\text{OFDM}}}  $$

**Fig. 5:**
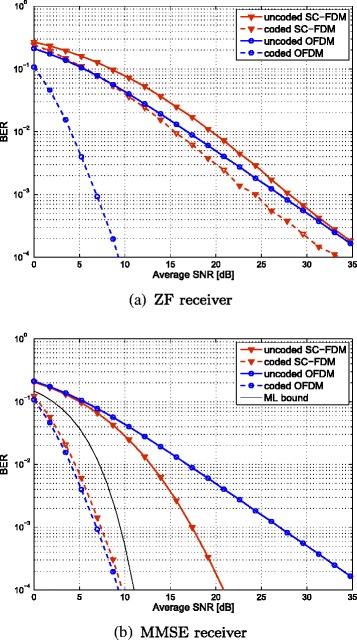
BER comparison between OFDM and SC-FDM for a SISO PedB channel with 5 MHz bandwidth and fixed CQI=4 transmission

Equality in Eq. (15) holds if and only if the channel is frequency flat. The difference between the harmonic mean and the arithmetic mean gets increasingly pronounced, the more selective the channel becomes. We therefore expect the (uncoded) BER of SC-FDM and ZF equalization to perform worse than OFDM, which is also validated by simulations. The BER simulations were carried out with CQI=4 on a PedB channel [34]. This modulation and coding scheme (MCS) employs 4-QAM and has an effective code-rate of 0.3008. As expected, the BER performance of SC-FDM is worse than OFDM, both shown in Fig. 5a in solid lines. Due to the spreading, SC-FDM already expends all channel diversity and coding does not increase the SNR slope of the BER curve. This manifests in an almost parallel shift of the BER curve for SC-FDM, as visual in Fig. 5a in red dashed lines. None exploited diversity allows coded OFDM to increase the BER slope considerably, cf. Fig. 5a blue dashed line.

The MMSE SINR expression is less intuitive and for the purpose of comparison, similar mathematical transformations as in [35] and [23] are required to arrive at

(16) $$  {\fontsize{8.4pt}{6pt}\begin{aligned} {}\text{SINR}_{\text{MMSE}}^{\text{SC-FDM}} &= \frac{{\sigma_{x}^{2}}}{{\sigma_{n}^{2}}} \frac{1-\frac{{\sigma_{n}^{2}}}{{\sigma_{x}^{2}}} \frac{1}{N_{\text{SC}}} \sum\limits_{k=1}^{N_{\text{SC}}} \frac{1}{\frac{{\sigma_{n}^{2}}}{{\sigma_{x}^{2}}}+|\boldsymbol{H}_{k}|^{2}} }{\frac{1}{N_{\text{SC}}}\sum\limits_{k=1}^{N_{\text{SC}}} \frac{1}{\frac{{\sigma_{n}^{2}}}{{\sigma_{x}^{2}}}+|\boldsymbol{H}_{k}|^{2}}} \\ &=\frac{{\sigma_{x}^{2}}}{{\sigma_{n}^{2}}}\left(\frac{1}{\frac{1}{N_{\text{SC}}}\sum\limits_{k=1}^{N_{\text{SC}}} \frac{1}{\frac{{\sigma_{n}^{2}}}{{\sigma_{x}^{2}}}+|\boldsymbol{H}_{k}|^{2}}} -\frac{{\sigma_{n}^{2}}}{{\sigma_{x}^{2}}} \right)~. \end{aligned}}  $$

The detailed derivation is shown in the Appendix. The denominator of Eq. (16) is regularized and less sensitive to spectral notches.

An upper bound on the SINR can be obtained via the maximum of the transfer function ***H***_*k*_

(17) $$\begin{array}{@{}rcl@{}} \text{SINR}_{\text{MMSE}}^{\text{SC-FDM}} &\le& \frac{{\sigma_{x}^{2}}}{{\sigma_{n}^{2}}}\left(\frac{1}{ \frac{1}{ \;\; \frac{{\sigma_{n}^{2}}}{{\sigma_{x}^{2}}}+\max_{k}|\boldsymbol{H}_{k}|^{2}} \;\;} -\frac{{\sigma_{n}^{2}}}{{\sigma_{x}^{2}}} \right) \\ &=& \frac{{\sigma_{x}^{2}}}{{\sigma_{n}^{2}}} \max_{k}|\boldsymbol{H}_{k}|^{2}  ~. \end{array} $$

In the low SNR regime $\frac {{\sigma _{n}^{2}}}{{\sigma _{x}^{2}}} \gg |\boldsymbol {H}_{k}|^{2}$, this bound becomes tight. The higher the inverse SNR $\frac {{\sigma _{n}^{2}}}{{\sigma _{x}^{2}}}$ in relation to the maximum of the transfer function, the tighter the bound becomes. The average OFDM SNR can never be larger to its maximum entry and is only equal for frequency flat channels. At low SNR, a lower BER is thus expected. Again, this presumption is validated by our simulation, showing that the uncoded BER is lower for SC-FDM as for ODFM, cf. Fig. 5b in solid lines. Although the uncoded BER shows superior performance, the coded BER is lower for OFDM due to the coding gains stemming from channel diversity, cf. Fig. 5b dashed lines.

A bound for the maximum likelihood (ML) detection performance was derived in [36]. As the bandwidth increases, the slope of the BER curve achieved with MMSE receivers tends to the slope of ML detection, demonstrating the full exploitation of channel diversity by the MMSE equalizer, cf. Fig. 5b black line.

## Link adaptation

In the previous section, we investigated BER performance of OFDM and SC-FDM transmission with different channel models and receivers. We observed significant BER degradation of SC-FDM as compared to OFDM when ZF detection is employed, whereas coded BER is very similar when MMSE detection is used. In this section, we evaluate how such BER differences impact the actual throughput performance of LTE-A uplink when transmission rate adaptation is employed. We first consider ideal rate adaptation and compare SC-FDM transmission to OFDM with ZF and MMSE receivers. Then, we extend our single-user MIMO CSI feedback algorithms proposed for LTE downlink in [37] to LTE uplink and evaluate their performance comparing to the throughput bounds developed in [24]. We also highlight some important basic differences between link adaptation in LTE up- and downlink transmissions.

### Performance with ideal rate adaptation

**Fig. 6:**
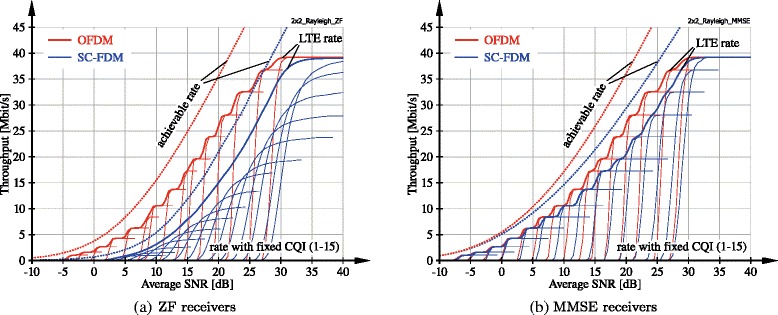
Throughput comparison of OFDM and SC-FDM with rate adaptation and 2×2 Rayleigh fading channels of 5-MHz bandwidth

As demonstrated in the previous section, SC-FDM provides a significant advantage in terms of PAPR over OFDM, thus relaxing linearity requirements of radio frequency power amplifiers for user equipments. Yet, this comes at the cost of coded BER degradation since channel diversity is lost and the performance is mostly dominated by the weakest subcarrier of a user, especially with ZF receivers; c.f. (12). This diversity loss cannot be recovered by forward-error-correction channel coding, since the DFT-spreading applied with SC-FDM effectively causes an averaging over SINR observed on all scheduled subcarriers according to (??). As a consequence, SC-FDM transmission over frequency selective channels achieves worse throughput than OFDM. This is demonstrated in Fig. 6, where we cross-compare the achievable rate, as defined in (18) and (19), and the actual throughput of SC-FDM and OFDM transmission as obtained by the Vienna LTE-A Uplink Simulator. We consider single-user transmission over 5 MHz bandwidth assuming *N*_T_=*N*_R_=2 antennas at the user and the base station and *L*=2 spatial layers. The precoder is selected as a scaled identity matrix: $\boldsymbol {W} = 1/\sqrt {L}\, \boldsymbol {I}_{L}$. We consider transmission over independent and identically distributed frequency-selective Rayleigh fading channels, emphasizing the difference between OFDM and SC-FDM. The achievable rate in bits per OFDM/SCFDM symbol with Gaussian signalling and equal power allocation over subcarriers and spatial layers is calculated as

(18) $$ R^{\text{OFDM}} = \sum_{k = 1}^{N_{\text{SC}}} \sum_{l = 1}^{L} \text{log}_{2}\left(1 + \left[\boldsymbol{\operatorname{{SINR}}}^{\text{OFDM},\;(l)}\right]_{k} \right),  $$

(19) $$ R^{\text{SC-FDM}} = N_{\text{SC}} \sum_{l = 1}^{L} \text{log}_{2}\left(1 + {\operatorname{{SINR}}}^{\text{SC-FDM},\;(l)} \right),  $$

with the receiver-specific post-de-spreading (post-equalization) SINRs from (??) and (8), respectively.

We observe a significant loss of achievable rate of SC-FDM transmission compared to OFDM in Fig. 6, which is especially pronounced with ZF receivers due to noise enhancement. In Fig. 6, we also show the actual rate achieved by LTE uplink SC-FDM transmission with ideal rate adaptation and compare to the performance obtained by OFDM transmission; the corresponding curves are denoted by *LTE rate*. We determine the performance of ideal rate adaptation by simulating all possible transmission rates, corresponding to CQI1 to CQI15, and selecting at each subframe the largest rate that achieves error free transmission. The figure also shows the throughput of the individual CQIs. We observe a gap between the LTE throughput with OFDM and SC-FDM that is similar to the gap in terms of achievable rate. Notice that the performance loss with MMSE receivers is significantly smaller than with ZF detection, since MMSE avoids excessive noise enhancement.

We also observe in Fig. 6a that the gain achieved by instantaneous rate adaptation, as compared to rate adaptation based on the long-term average SNR, is much larger for ZF SC-FDM than for ZF OFDM; this is evident from the distance between the curves with rate adaptation (LTE rate) and the curves with *fixed CQI*. The reason for this behaviour is that the SNR of ZF SC-FDM shows strong variability around its means, since it is dominated by the worst-case per-subcarrier SNR according to (12); the average SNR over subcarriers of ZF OFDM, however, approximately coincides with its mean value. This implies that the optimal CQI of ZF SC-FDM can vary significantly in-between subframes, as reflected by the large average SNR variation required to increase the rate with fixed CQI from zero to its respective maximum. Yet, for ZF OFDM, the throughput of the individual CQIs follows almost a step function; hence, rate adaptation can be based on the long-term average SNR without substantial performance degradation.^5^

In case *N*_R_>*L*, we can easily estimate the achievable rate of SC-FDM transmission: The per-layer SNR with ZF receivers is governed by the harmonic mean of the channel responses on the individual subcarriers, similar to (12)

(20) $$ {\operatorname{{SNR}}}_{\text{ZF}}^{\text{SC--FDM},\;(l)} = \frac{{\sigma_{x}^{2}}/{\sigma_{n}^{2}}}{\frac{1}{N_{\text{SC}}} \sum_{k = 1}^{N_{\text{SC}}} \left[\!\left(\! \left(\boldsymbol{H}_{k} \boldsymbol{W}\right)^{\mathrm{H}} \! \left(\boldsymbol{H}_{k} \boldsymbol{W}\right)\!\right)^{{}_{-1}}\right]_{l,l}},   $$

with $\boldsymbol {H}_{k} \in \mathbb {C}^{N_{\mathrm {R}} \times N_{\mathrm {T}}}$ denoting the OFDM channel matrix on subcarrier *k*. Assuming constant precoding and semi-correlated Rayleigh fading

(21) $$ \boldsymbol{H}_{k} = \tilde{\boldsymbol{H}}_{k} \boldsymbol{C}_{T}^{\frac12},\quad \left[\tilde{\boldsymbol{H}}_{k}\right]_{i,j} \sim \mathcal{CN}\left\{0,1\right\},  $$

with $\boldsymbol {C}_{T} \in \mathbb {C}^{N_{\mathrm {T}} \times N_{\mathrm {T}}}$ determining the spatial correlation at the user equipment side, the matrix in the denominator of (20) follows a complex inverse Wishart distribution with *N*_R_ degrees of freedom and scale matrix $\boldsymbol {C} = \left (\boldsymbol {W}^{\mathrm {H}} \boldsymbol {C}_{T} \boldsymbol {W}\right)^{-1}$

(22) $$ \overline{\boldsymbol{H}} = \left(\left(\boldsymbol{H}_{k} \boldsymbol{W}\right)^{\mathrm{H}} \left(\boldsymbol{H}_{k} \boldsymbol{W}\right)\right)^{-1} \sim \mathcal{CW}_{L}^{-1}\left\{N_{\mathrm{R}},\boldsymbol{C}\right\}.  $$

Letting $N_{\text {SC}} \rightarrow \infty $, we can replace the term in the denominator of (20) with its expected value

(23) $$ \frac{1}{N_{\text{SC}}} \sum_{k = 1}^{N_{\text{SC}}} \left[\;\overline{\boldsymbol{H}}\;\right]_{l,l} \overset{N_{\text{SC}} \rightarrow \infty}{\longrightarrow} \mathbb{E}\left(\left[\;\overline{\boldsymbol{H}}\;\right]_{l,l} \right).  $$

This expected value only exists in case *N*_R_>*L* [38]. For *N*_R_=*L*, the diagonal elements of $\overline {\boldsymbol {H}}$ follow a heavy-tailed inverted Gamma distribution [39, 40] with non-finite first moment. Yet, for *N*_R_>*L*, which is a common situation in cellular networks since the base station is mostly equipped with far more antennas than the users, the expected value is

(24) $$ \mathbb{E}\left(\left[\;\overline{\boldsymbol{H}}\;\right]_{l,l} \right) = \frac{1}{N_{\mathrm{R}} - L} \left[\boldsymbol{C}\right]_{l,l}.  $$

Hence, we can estimate the achievable rate of SC-FDMA transmission over semi-correlated Rayleigh fading channels

(25) $$\begin{array}{*{20}l} &R^{\text{SC-FDM}} \!\approx\! N_{\text{SC}} \sum_{l = 1}^{L} \text{log}_{2}\left(1 \,+\, \frac{{\sigma_{x}^{2}}/{\sigma_{n}^{2}}}{\left[\boldsymbol{C}\right]_{l,l}} (N_{\mathrm{R}}\,-\,L) \right)  \end{array} $$

(26) $$\begin{array}{*{20}l} &\approx N_{\text{SC}} L \left[\text{log}_{2}\left(\frac{{\sigma_{x}^{2}}/{\sigma_{n}^{2}}}{\left(\prod_{l = 1}^{L} \left[\boldsymbol{C}\right]_{l,l}\right)^{1/L}} \right) + \text{log}_{2}\left(N_{\mathrm{R}}\,-\,L\right)\right].  \end{array} $$

Here, (26) resembles the high SNR approximation of the achievable rate of OFDM transmission with ZF detection as proposed in ([41] Eq. (14)); even more, for fixed *L* and letting *N*_R_ grow to infinity, (26) and ([41] Eq. (14)) tend to the same limit, due to channel hardening on each subcarrier with growing number of receive antennas.

**Fig. 7:**
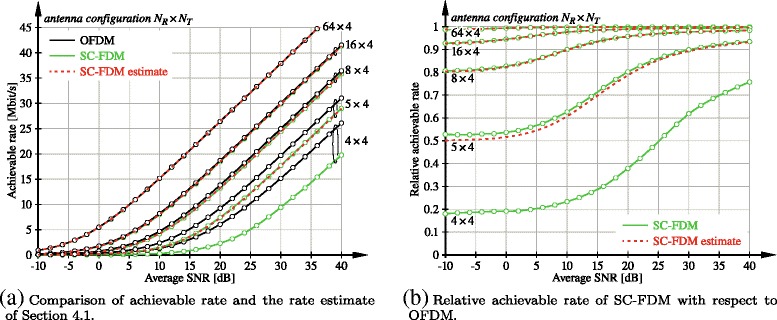
Achievable rate of OFDM and SC-FDM with ZF equalizers and growing number of receive antennas at fixed number of streams *L*=4

In Fig. 7, we investigate the performance of the rate estimate (25) for *N*_T_=*L*=4 and varying number of receive antennas. We assume $\boldsymbol {W} = 1/\sqrt {L}\, \boldsymbol {I}_{L}$ and

$$ \boldsymbol{C}_{T} = \left[\begin{array}{cccc} 1 & 0.9 & \ldots & 0.9 \\ 0.9 & \ddots & & \vdots \\ \vdots & & & 0.9 \\ 0.9 & \ldots & 0.9 & 1\end{array}\right],   $$

and consider the smallest LTE bandwidth of *N*_SC_=72 subcarriers. We observe that the proposed estimate performs very well even at this small bandwidth; notice, though, that a more realistic channel model with correlation over subcarriers may require larger bandwidth to validate the proposed estimate. Figure 7 also confirms the observation that single-user MIMO OFDM and SC-FDM with ZF detectors tend to the same limiting performance with increasing number of receive antennas.

This statement, however, will not hold true if the total number of layers grows proportionally with the number of receive antennas. For example, multi-user MIMO transmission with ZF equalization and single-antenna users achieves only a diversity order of *N*_R_−*L*+1 [42], with *L* denoting the total number of layers being equal to the number of spatially multiplexed users. Hence, if *L* scales proportionally with *N*_R_, channel hardening on each subcarrier will not occur and thus the performance of OFDM and SC-FDM will not coincide.

### Performance with realistic link adaptation

Instantaneous rate adaptation is an important tool for exploiting diversity of the wireless channel in LTE, by adjusting the transmission rate according to the current channel quality experienced by a user. LTE specifies a set of 15 different MCSs; the selected MCS is signalled by the CQI.

LTE additionally supports spatial link adaptation by means of codebook-based precoding with variable transmission rank. With this method, the precoding matrix $\boldsymbol {W} \in \mathbb {C}^{N_{\mathrm {T}} \times L}$ satisfying $\boldsymbol {W}^{\mathrm {H}} \boldsymbol {W} = 1/L\, \boldsymbol {I}_{L}$ is selected from a standard-defined codebook $\mathcal {W}_{L}$ of scaled semi-unitary matrices; furthermore, the number of spatial layers *L* can be adjusted to achieve a favourable trade-off between beamforming and spatial multiplexing. The selected precoder and transmission rank are signalled, employing the PMI and the RI. In single-user MIMO LTE uplink transmission, the same precoder is applied on all RBs that are assigned to a specific user, whereas frequency-selective precoding is supported in LTE downlink.

There is a basic difference between the utilization of CQI, PMI and RI in up- and downlink directions of frequency division duplex (FDD) systems. In the downlink, the base station is reliant on CSI feedback from the users for link adaptation and multi-user scheduling [43], since channel reciprocity cannot be exploited in FDD. CQI, PMI and RI can be employed to convey such CSI from the users to the base station via dedicated feedback channels [44]. In the uplink, on the other hand, the base station can by itself determine CSI exploiting the sounding reference signals (SRSs) transmitted by the users. In this case, CQI, PMI and RI are employed by the base station to convey to the users its decision on link adaptation that has to be applied by the users during uplink transmission.

In principal, link adaptation must be jointly optimized with multi-user scheduling to optimize the performance of the system, since the effective SC-FDM SINR (and thus the rate) of a user depends on the assigned RBs according to (??). For reasons of computational complexity, however, we assume that the multi-user schedule is already fixed and determine link adaptation parameters based on this resource allocation. We modify the approach proposed in [37] for LTE downlink transmission to determine the link adaptation parameters in four steps:

1. Determine the optimal precoder for each transmission rank $L \leq \min \left (N_{\mathrm {T}},N_{\mathrm {R}}\right)$ by maximizing transmission rate
  (27) $$ \hat{\boldsymbol{W}}(L) = {\underset{\boldsymbol{W} \in \mathcal{W}_{L}}{\text{arg\,max}}}\, \sum_{l = 1}^{L} f\left({\operatorname{{SINR}}}^{\text{SC-FDM},\;(l)} \left(\boldsymbol{W} \right)\right).  $$
  Here, function *f*(·) maps SINR to rate; this could be either an analytical mapping, such as (19), or a mapping table representing the actual performance of LTE. In our simulations, we employ the bit interleaved coded-modulation (BICM) capacity as proposed in [37], since LTE is based on a BICM architecture.
2. Determine the optimal LTE transmission rates per layer for each *L* and $\hat {\boldsymbol {W}}(L)$. We employ a target block error ratio (BLER) mapping in our simulations to determine the highest rate that achieves BLER≤0.1.
3. Select the transmission rank $\hat {L}$ that maximizes the sum rate over spatial layers, utilizing the LTE transmission rates determined above.
4. Set the RI and PMI according to $\hat {L}$ and $\hat {\boldsymbol {W}}(L)$, respectively, and set the pCQI conforming to the corresponding LTE transmission rates.

**Fig. 8:**
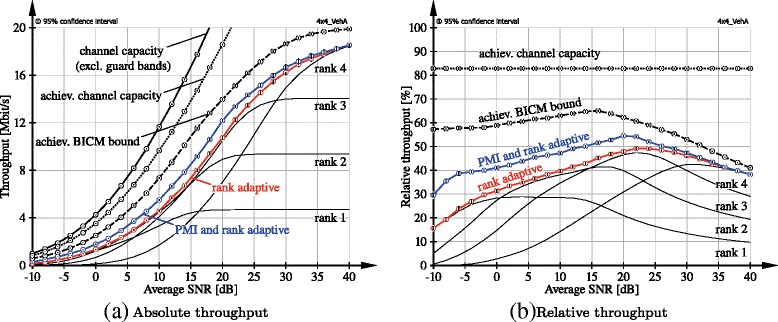
Absolute and relative throughput of LTE uplink transmission over 4×4 VehA channels of 1.4-MHz bandwidth employing rate adaptation. We compare the performance of fixed rank, rank adaptive and PMI + rank adaptive transmission to the performance bounds proposed in [24]

In Fig. 8, we evaluate the performance of single-user MIMO LTE uplink transmission over *N*_T_=*N*_R_=4 antennas with link adaptation, 1.4 MHz system bandwidth and ZF receiver. We do not consider signalling delays between the base station and the user. We employ the VehA channel model [34] and compare the absolute and relative (to channel capacity) throughput to the performance bounds proposed in [24].^6^*Channel capacity* is obtained by applying singular value decomposition (SVD)-based transceivers and water-filling power allocation over subcarriers and spatial streams. Notice that we do not account for guard band and CP overheads when calculating the channel capacity; that is, we only consider subcarriers that are available for data transmission. The *achievable channel capacity* takes overhead for pilot symbols (DMRS and SRS) into account, corresponding to a loss of 16.7 % in our simulation. The *achievable BICM bound* additionally accounts for equal power allocation, codebook-based precoding and ZF detection as well as the applied BICM architecture as detailed in [24].

The performance of LTE uplink transmission with full link adaptation (*PMI and rank adaptive*) is similar to the achievable BICM bound but shifted by approximately 3 dB. Notice that the saturation value is not the same because the highest CQI of LTE achieves 5.55 bit/channel use, whereas the BICM bound saturates at 6 bit/channel use. We also show the performance of LTE uplink when restricted to fixed precoding (*rank adaptive*) and fixed rank transmission (*ranks 1, 2, 3, 4*). We observe that rank adaptive transmission even outperforms the envelope of the fixed rank transmission curves, since instantaneous rank adaptation selects the optimal rank in each subframe independently. In terms of relative throughput, we see that LTE uplink with ZF receivers achieves around 40–50 % of channel capacity; remember, though, that this does not include CP and guard band overheads.

## Reference symbols

**Fig. 9:**
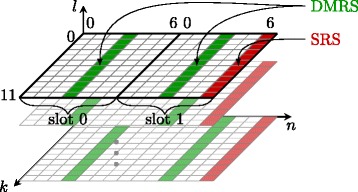
The LTE-A uplink reference symbol allocation in two slots (one subframe)

In LTE uplink, two types of reference signals are standardized. For CE and coherent detection, DMRS are exploited, while SRS are employed for channel sounding to enable frequency selective scheduling. For the purpose of CE, we will consider DMRS only. The reference symbols are defined in [1] and are explained in more detail in [45, 46]. As shown in Fig. 9, DMRS are multiplexed in the resource grid at OFDM symbol time *n*=3 in every slot. In a physical uplink shared channel (PUSCH) transmission of the LTE-A uplink, a DMRS occupies all scheduled subcarriers. We assume that the user is assigned to all *N*_SC_ subcarriers starting at 0, i.e., *k*∈{0,1,…,*N*_SC_−1}. We denote the Zadoff-Chu (ZC) base sequence on *N*_SC_ subcarriers for one slot by $\boldsymbol {\bar {r}} \in \mathbb {C}^{N_{\text {SC}} \times 1}$. The base sequences $\boldsymbol {\bar {r}}$ are complex exponential sequences lying on the unit circle fulfilling

(28) $$ \left| \left[ \boldsymbol{\bar{r}} \right]_{k} \right| = 1~.   $$

In LTE-A, the DMRS of different transmission layers in the same slot are orthogonal in terms of frequency domain code division multiplexing (FD-CDM) [45]. This is obtained by cyclically shifting the base sequence. Similar to [47], DMRS on layer *l* for one slot are given by

(29) $$ \boldsymbol{R}^{(l)}= \text{Diag} \left(\boldsymbol{r}^{(l)} \right) = \boldsymbol{T}^{(l)} \text{Diag}{\left(\boldsymbol{\bar{r}}\right)}~,   $$

with the cyclic shift operator

(30) $$ \boldsymbol{T}^{(l)}=\text{Diag}\left(e^{j0},\hdots,e^{j\alpha_{l} k},\hdots,e^{j\alpha_{l} (N_{\text{SC}}-1)}\right)~,   $$

and the layer dependent cyclic shift *α*_*l*_. We further conclude from (28) to (30) that (***R***^(*l*)^)^*H*^=(***R***^(*l*)^)^−1^ which implies $\left (\boldsymbol {R}^{(l)}\right)^{H} \boldsymbol {R}^{(l)} = \boldsymbol {I}_{N_{\text {SC}}}$. Exploiting (28), the product of two DMRS from layers *l* and *u* with *l*,*u*∈{1,…,*L*} becomes

(31) $$\begin{array}{@{}rcl@{}} \left(\boldsymbol{R}^{(l)}\right)^{H} \boldsymbol{R}^{(u)} &=&\left(\boldsymbol{T}^{(l)} \right)^{H} \boldsymbol{T}^{(u)} \text{Diag}~{\left(\boldsymbol{\bar{r}}\right)}^{H} \text{Diag}~{\left(\boldsymbol{\bar{r}}\right)}\\ &=& \text{Diag} \left(e^{j0} \;\hdots\; e^{j\Delta\alpha k} \;\hdots\; e^{j\Delta\alpha (N_{\text{SC}}-1)} \right) \boldsymbol{I} ~, \end{array} $$

with *Δα*=*α*_*u*_−*α*_*l*_ being the cyclic phase shift between DMRS of two different spatial layers. The FD-CDM orthogonality can therefore be exploited as

(32) $$ \text{trace}\left(\! \left(\boldsymbol{R}^{(u)}\right)^{H} \boldsymbol{R}^{(l)} \right) \! = \! \left(\boldsymbol{r}^{(u)}\right)^{H} \boldsymbol{r}^{(l)} \! = \left\{\begin{array}{ll} N_{\text{SC}} &\text{for}~u=l \\ 0 &\text{for}~u\neq l ~. \end{array} \right.  $$

After transmission over a frequency selective channel, this orthogonality has to be exploited to separate all effective MIMO channels at the receiver.

## Channel estimation

For channel estimation we exploit the system model only at symbol times, where reference signals are allocated. For a normal CP length, this is the 4th symbol in each slot, i.e. *n*=3 as shown in Fig. 9. Since we estimate the channel only at this single symbol time per slot, interpolation in time has to be carried out to obtain channel estimates for the whole resource grid. The effects of interpolation will be studied in Section 7. As illustrated in Fig. 2, the DMRS are added after DFT spreading, right before precoding. As the channel estimation takes place after the receiver’s DFT, just before equalization, the system model for CE amounts to an OFDM system. The system model (1) therefore reads as

(33) $$  \boldsymbol{y} = \boldsymbol{H}_{\text{eff}} \boldsymbol{r} + \boldsymbol{n'} ~,  $$

with (pre-equalization) noise

(34) $$  \boldsymbol{n'} = \left(\boldsymbol{I}_{N_{R}} \otimes \boldsymbol{M}^{H} \boldsymbol{D}_{N_{\text{FFT}}} \boldsymbol{P}_{\text{remCP}}\right) \boldsymbol{n} ~,  $$

and the stacked vector ***r*** consisting of DMRS $\boldsymbol {r}^{(l)} \in \mathbb {C}^{N_{\text {SC}} \times 1}$ from all active spatial layers $l\in \lbrace 1,\dots,L \rbrace $, i.e. $\boldsymbol {r} = \left (\left (\boldsymbol {r}^{(1)}\right)^{T},\dots,\left (\boldsymbol {r}^{(L)}\right)^{T}\right)^{T}$. To consider the received signal separately for each receive antenna *i*, we can select the according part from ***y*** by left multiplying with the selector matrix ***S***^(*i*)^ from (6). The received signal ***y***^(*i*)^=***S***^(*i*)^***y*** on antenna *i* is given by

(35) $$\begin{array}{*{20}l}  \boldsymbol{y}^{(i)} = & \left(\boldsymbol{H}_{\text{eff}}^{(i,1)},\dots, \boldsymbol{H}_{\text{eff}}^{(i,L)} \right) \boldsymbol{r} + \boldsymbol{n'}^{(i)} \\ = & \sum_{l=1}^{L} \boldsymbol{H}_{\text{eff}}^{(i,l)} \boldsymbol{r}^{(l)} + \boldsymbol{n'}^{(i)} ~, \notag \end{array} $$

with the pre-equalization noise ***n***^***′***^^(*i*)^=***S***^(*i*)^***n***^***′***^ on receive antenna *i* and $\boldsymbol {H}^{(i,l)}_{\text {eff}} = \boldsymbol {S}^{(i)} \boldsymbol {H}_{\text {eff}} \left (\boldsymbol {S}^{(l)}\right)^{T}$ being the (*i*,*l*)^th^ block of ***H***_eff_. Since $\boldsymbol {H}^{(i,l)}_{\text {eff}}$ is diagonal, we exploit the relations $\boldsymbol {R}^{(l)} = \text {Diag} \left (\boldsymbol {r}^{(l)} \right)$ and $\boldsymbol {h}_{\text {eff}}^{(i,l)} = \text {diag} \left (\boldsymbol {H}_{\text {eff}}^{(i,l)} \right)$ to estimate a channel vector rather than a matrix and rearrange terms in (35) leading to

(36) $$\begin{array}{*{20}l}  \boldsymbol{y}^{(i)} = & \sum_{l=1}^{L} \boldsymbol{R}^{(l)} \boldsymbol{h}_{\text{eff}}^{(i,l)} + \boldsymbol{n'}^{(i)} \\ = & \underbrace{\left(\boldsymbol{R}^{(1)},\dots, \boldsymbol{R}^{(L)} \right)}_{\boldsymbol{R}} \boldsymbol{h}_{\text{eff}}^{(i)} + \boldsymbol{n'}^{(i)} ~, \notag \end{array} $$

with the stacked vector $\boldsymbol {h}_{\text {eff}}^{(i)} = \left (\left (\boldsymbol {h}_{\text {eff}}^{(i,1)}\right)^{T},\dots,\left (\boldsymbol {h}_{\text {eff}}^{(i,L)}\right)^{T}\right)^{T}$ of all effective channels from *L* active layers to receive antenna *i* for which we will drop the subscript in the following.

### Minimum mean square error estimation

First, we present a MMSE estimator where we exploit (36) and estimate the stacked vector ***h***^(*i*)^ consisting of effective channels from all *L* active layers to receive antenna *i*. The MMSE CE for receive antenna *i* is given by

(37) $$ \boldsymbol{\hat{h}}^{(i)}_{\text{MMSE}} = \underset{\boldsymbol{\hat{h}}^{(i)}}{\text{arg min}}\, \mathbb{E} \left\lbrace \big\| \boldsymbol{\hat{h}}^{(i)} - \boldsymbol{h}^{(i)} \big\|_{2}^{2} \right\rbrace ~,   $$

which leads to the well-known solution [48]

(38) $$  \boldsymbol{\hat{h}}^{(i)}_{\text{MMSE}} = \left(\sigma_{\boldsymbol{n}^{(i)}}^{2} \boldsymbol{C}_{\boldsymbol{h}^{(i)}}^{-1} + \boldsymbol{R}^{H} \boldsymbol{R}\right)^{-1} \boldsymbol{R}^{H} \boldsymbol{y}^{(i)} ~,  $$

with $\boldsymbol {C}_{\boldsymbol {h}^{(i)}} = \mathbb {E} \lbrace \boldsymbol {h}^{(i)} \boldsymbol {h}^{(i)H}\rbrace $.

### Correlation-based estimation

As a low complexity approach, we correlate (matched filter) the received signal with the reference symbol of layer *l* to obtain a channel estimate for the effective channel ***h***^(*i*,*l*)^ from layer *l* to receive antenna *i*

(39) $$ \boldsymbol{\tilde{h}}^{(i,l)} = \left(\boldsymbol{R}^{(l)}\right)^{H} \boldsymbol{y}^{(i)} ~.   $$

In fact, this correlation approach is optimum in a least squares (LS) sense

(40) $$ \boldsymbol{h}_{\text{LS}}^{(i)}=\underset{\boldsymbol{h}}{\text{arg min}}\, \| \boldsymbol{y}^{(i)}-\boldsymbol{R}\boldsymbol{h} \|_{2}=\left(\underbrace{\boldsymbol{R}\boldsymbol{R}^{H}}_{L\boldsymbol{I}} \right)^{-1} \boldsymbol{R}^{H} \boldsymbol{y}^{(i)}~.  $$

Inserting our system model (36) and exploiting (31), we obtain

(41) $$\begin{array}{@{}rcl@{}}  \boldsymbol{\tilde{h}}^{(i,l)} = & \left(\boldsymbol{R}^{(l)}\right)^{H} \sum_{u=1}^{L} \boldsymbol{R}^{(u)} \boldsymbol{h}^{(i,u)} + \left(\boldsymbol{R}^{(l)}\right)^{H} \boldsymbol{n'}^{(i)} \notag\\ = & \boldsymbol{h}^{(i,l)} + \underbrace{\sum_{\substack{u=1\\ u\neq l}}^{L} \left(\boldsymbol{T}^{(l)}\right)^{H} \boldsymbol{T}^{(u)} \boldsymbol{h}^{(i,u)}}_{\text{inter-layer interference}} + \; \boldsymbol{\tilde{n}}^{(i)} ~. \end{array} $$

Here, $\boldsymbol {\tilde {n}}^{(i)}$ has the same distribution as ***n***^***′***^^(*i*)^ since (***R***^(*l*)^)^*H*^ is unitary and introduces phase changes only, cf. (29). Due to the allocation of DMRS on the same time and frequency resources on different spatial layers, the initial estimate $\boldsymbol {\tilde {h}}^{(i,l)}$ of one effective MIMO channel actually consists of a superposition of all *L* effective MIMO channels to receive antenna *i*. The unintentional contributions in (41), from layers *u*≠*l* are inter-layer interference, making it unsuited as initial estimate for coherent detection. Different methods to separate the different effective MIMO channels in (41) will be presented in the following.

#### DFT-based channel estimation

A well-known approach for CE in LTE-A uplink is DFT-based estimation [46], which aims to separate the MIMO channels contributing to (41) in time domain. For this, the individual cyclic shift of each DMRS is exploited. Applying a DFT on the receive signal, the individual phase shifts will translate into shifts in time domain. This makes a separation of channel impulse response (CIR)s from different MIMO channels possible by windowing. In our simulator, we implemented a DFT-based estimator as in [49] or [47].

#### Averaging

For physically meaningful channels, neighbouring subcarriers will be correlated within the coherence bandwidth [50]. We utilize this property and exploit the DMRS structure to perform frequency domain CE. As explained in [25], applying a sliding averaging on the initial estimate $\boldsymbol {\tilde {h}}^{(i,l)}$ from (41) over $\bar {\gamma }$ adjacent subcarriers ($\bar {\gamma }$ equals 1,2,4,4 for *L* equals 1, 2, 3, 4, respectively) cancels the inter-layer interference, assuming the channel to be frequency flat on these $\bar {\gamma }$ consecutive subcarriers. The sliding average is given by

(42) $$ \left[ \boldsymbol{\hat{h}}_{\text{SAV}}^{(i,l)} \right]_{k} = \frac{1}{\bar{\gamma}^{2}} \sum_{t= k-\bar{\gamma} +1}^{k} \sum_{j=t}^{t+\bar{\gamma}-1} \left[ \boldsymbol{\tilde{h}}^{(i,l)} \right]_{j} ~,   $$

for $\bar {\gamma } \leq k \leq N_{\text {SC}}-\bar {\gamma } +1$. The second sum describes the averaging of $\bar {\gamma }$ elements while the first sum describes the shift of this averaging window.

#### Quadratic smoothing

Another method exploiting channel correlations to estimate the channel in frequency domain is quadratic smoothing (QS). This scheme cannot remove the inter-layer interference entirely, which manifests in a higher error floor, but shows improved performance at lower SNR in return. As explained in [25], this estimation method, exploiting the smoothing matrix ***Q*** and a smoothing factor *γ*, is given by

(43) $$\begin{array}{*{20}l}  \boldsymbol{\hat{h}}_{\text{QS}}^{(i,l)} = \left(\boldsymbol{I}_{N_{\text{SC}}L} + \lambda \boldsymbol{Q}^{H}\boldsymbol{Q} \right)^{-1} \underbrace{\left(\boldsymbol{{R}}^{(l)}\right)^{H} \boldsymbol{{y}}^{(i)}}_{\boldsymbol{\tilde{h}}^{(i,l)}} ~. \end{array} $$

Similar to (42), this can be interpreted as another way to cope with the inter-layer interference in (41) by post processing. This method does not use the DMRS structure explicitly but suppresses the interference by smoothing. It is therefore not able to cancel the complete inter-layer interference but shows an improved performance at low SNR.

### MSE and BER comparison

**Fig. 10:**
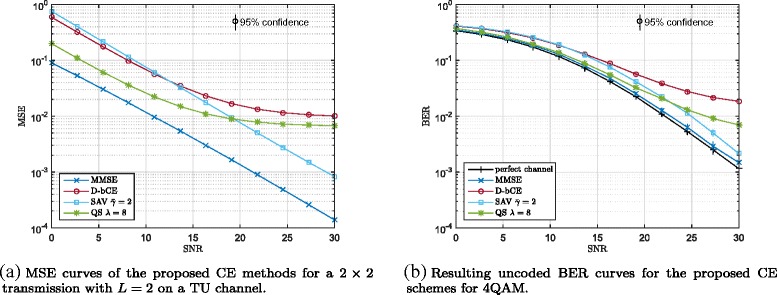
Channel estimation performance comparison for block fading

We assume a single user 2×2 MIMO transmission with *N*_SC_=72 subarriers, a fixed number of layers *L*=2 and a typical urban (TU) channel model [34] at zero speed. We perform a simulation with one-point extrapolation, cf. Section 7, and show the MSE curves of the proposed estimators in Fig. 10a. The DFT-based CE (*D-bCE*) shows the highest error flow of all estimators at high SNR while the *MMSE* estimator of course shows best performance over the whole SNR range. Compared to these two methods, the Sliding-Averaging estimator (42), denoted by *SAV*, encounters an 8-dB SNR penalty when compared to MMSE but comes closest to *MMSE* performance at high SNR. The quadratic smoothing estimation is denoted by *QS* and shows a significant improvement for low SNRs because it smooths over several observed channel coefficients. Quadratic smoothing performs uniformly better than *D-bC*E over the whole SNR range and comes close to 4 dB to MMSE at low SNR. The high error floor shows that QS is not able to cancel all the inter-layer interference.

In terms of BER performance, at high SNR, naturally the estimation method with the lowest MSE leads to the smallest BER. At low SNR, the difference in CE MSE translates into very small differences in BER, meaning, we cannot gain too much from a good low SNR MSE performance of QS or MMSE estimation. Considering estimation complexity and that MMSE as well as QS require prior channel knowledge, *SAV* estimation is a good complexity performance trade-off.

## Channel interpolation

Under fast fading conditions, additional effects influence the performance of LTE uplink transmissions. Doppler shifts degrade the SINR by introducing velocity dependent ICI [51] whereas the SINR increases with increasing subcarrier spacing. The subcarrier spacing of 15 kHz that is used in LTE makes transmissions quite robust against ICI. The impact of ICI becomes only evident at high velocities and high SNR. Figure 12b shows the BER for the case of perfect channel knowledge where the performance is only degraded by noise and ICI. At 200 km/h, the BER saturates due to ICI at high SNR whereas ICI mitigation techniques [52] show promising results to reduce this impact of ICI.

**Fig. 11:**
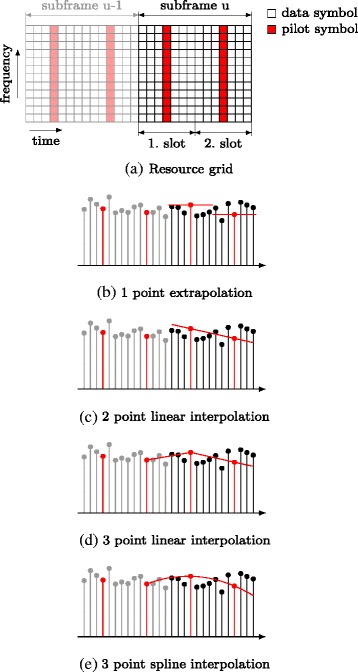
Channel interpolation techniques for the LTE-A uplink pilot pattern **a** using estimates from **b** the actual slot, **c** the actual subframe and **d**–**e** the actual and previous subframe

**Fig. 12:**
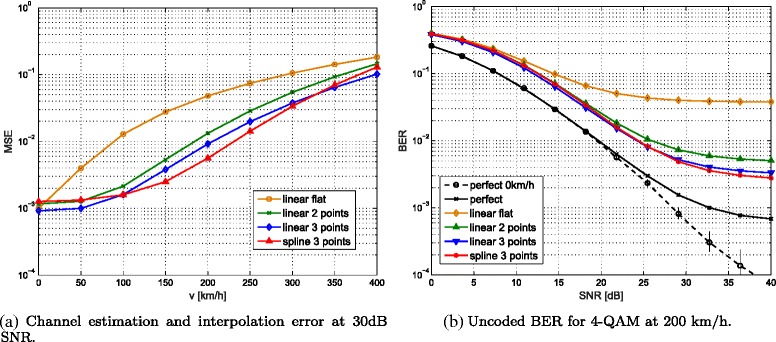
Comparison of channel interpolation techniques using different numbers of reference symbols and LS-SAV channel estimation

Another effect that hampers LTE transmissions at high velocities are temporal channel interpolation errors. While in the LTE downlink, the pattern used to multiplex data and reference symbols is a good trade-off between a small temporal and spectral spacing accounting for highly frequency selective channels and fast-fading channels and a rather small overhead, this is different in the uplink. As shown in Fig. 11a, uplink DMRSs occupy the whole subband. While there is no need for interpolation over frequency, the temporal spacing is about twice the spacing of the reference symbols in the downlink. Furthermore, if frequency hopping is performed, the number of adjacent pilots transmitted in the same subband is two for inter-subframe frequency hopping and only one for intra-subframe frequency hopping where frequency hopping is performed on a per-slot basis. Due to this special structure channel, interpolation in the LTE uplink is a challenging problem. Therefore, we investigated various channel interpolation techniques using a single, two or three consecutive pilot symbols. Figure 11b–e illustrates the channel interpolation techniques considered. The highest channel interpolation errors (Fig. 12a) are observed for *1 point extrapolation* where the channel estimate obtained in a certain slot is used to equalize the symbols within that slot and no interpolation is performed at all. The higher the number of pilots involved in channel interpolation, the lower the MSE gets. The results in terms of BER in Fig. 12b show a similar behaviour.

For a measurement-based comparison of interpolation techniques using channel estimates form both, the previous and the subsequent subframe, the reader is referred to [53].

## Future research questions

Until now our research efforts on the Vienna LTE-A Uplink Simulator have been concentrated on single links between user and base station, focusing on basic transceiver issues such as link adaptation and channel estimation. Our treatment of the link performance analysis is not considered complete. There are still important parameters to investigate, such as different forms of channel coding, enhanced channel estimation and detection [54, 55] and analysis of SC-FDM sensitivity to synchronization mismatch, similar to our downlink investigations [56].

In the future, our scope will shift to multi-user multi-base station scenarios, enabling on one hand exploitation of multi-user diversity in space, time and frequency and, on the other hand, consideration of interference in-between simultaneous transmissions from multiple base stations. Even though, for reasons of computational complexity, simulations will be confined to comparatively small scenarios containing some few base stations, we still expect to extract valuable performance indicators for coordinated multipoint reception schemes [57], accounting for practical constraints, such as, limited back-haul capacity.

We will address cross-layer multi-user scheduling, jointly optimizing multi-user resource allocation and per-user link adaptation; this is an intricate issue in LTE, due to the non-linear relationship between the resources assigned to a user and its corresponding SC-FDM SINR (??); we have already addressed this issue for the downlink in [43]. Multi-user scheduling, furthermore, has to find a favourable trade-off between transmission efficiency and fairness of resource allocation. We will extend existing downlink schedulers, which enable Pareto-efficient transmission with arbitrary fairness, to the uplink specifics and compare to other proposals, e.g. [58].

The realization of massive MIMO in LTE compliant systems is another highly important research topic, since it promises an order of magnitude network efficiency gains through spatial multiplexing of users [59–61]. Yet, many issues still need to be better understood and resolved to enable efficient massive MIMO transmission in practice. One important step towards reasonable performance investigation of massive antenna arrays is to employ realistic channel models, such as, the 3GPP three-dimensional channel model [62], which we plan to incorporate in future releases of our simulator.

## Conclusions

For an LTE-A uplink transmission model, we derived SINR expressions, both with and without DFT pre-spreading. We specialized these equations to ZF and MMSE receivers and showed that ZF performance is strongly affected by the worst subcarrier. Comparing the resulting BER we revealed that SC-FDM performance is generally inferior to OFDM and that applying MMSE equalization is crucial to get closer to OFDM performance.

Based on the system’s SINR, we analysed the achievable rate. We also introduced a method to estimate the SC-FDM rate for *N*_R_>*L*. Further, a possible calculation of LTE-A link adaptation parameters was proposed to achieve throughout close to performance bounds.

Lastly, we considered methods to gather CSI at the receiver. We compared the performance of various channel estimation and interpolation techniques. By incorporating the channel estimates of the previous subframe, we showed superior performance in terms of channel interpolation.

## Endnotes

^1^ Note that we use the symbol *n* as time index and the vector ***n*** for noise, the distinction should be clear from the context.

^2^ Within this paper we focus on a single user’s link performance. Multi user / multi basestation simulations are possible to perform, but come at very long simulation times. For sake of readability we use the non-standardized OFDM, SC-FDM notation in the remainder of this manuscript.

^3^ A multi-tap equalizer applied on the intralayer interference visual in Fig. 3c could possibly enhance the link performance.

^4^ The reduction to SISO is done to make our results comparable even to older frequency domain equalization (FDE) works, e.g., [63].

^5^ Notice, however, that instantaneous rate adaptation for ZF OFDM can be advantageous in case of frequency-correlated channels [44].

^6^ Notice that the simulation setup is the same as employed in [24] for the investigation of LTE downlink transmission, thus, facilitating the comparison of up- and downlink performance.

## Appendix

### General MIMO SC-FDMA SINR expression

The signal estimates are described via the input-output relationship in Eq. (1). We first slice out that part of ***K*** which acts on layer *l* by multiplying with the selector matrix ***S***^(*l*)^ from the left. As indicated in (1), the signal estimate consists of three contributions.

- $\hat {\boldsymbol {x}}_{\mathrm {s}}= \boldsymbol {S}^{(l)} \left (\boldsymbol {I}\odot \boldsymbol {K}\right) \boldsymbol {x}$
- $\hat {\boldsymbol {x}}_{\mathrm {i}}= \boldsymbol {S}^{(l)}\left (\boldsymbol {K}-\boldsymbol {I}\odot \boldsymbol {K}\right) \boldsymbol {x}$
- $\hat {\boldsymbol {x}}_{\mathrm {n}}=\boldsymbol {S}^{(l)} \tilde {\boldsymbol {n}} $

As ***x*** and $\tilde {\boldsymbol {n}}$ are zero mean random quantities, their power is described by means of the second moment. To calculate the second moments, we take out the diagonal elements of the respective covariance matrices of each contribution.

(44) $$\begin{array}{@{}rcl@{}} &{\text{SINR}_{l}^{\text{SC-FDM}}} = \\&\bigg[\left(\!\boldsymbol{I}\odot\mathbb{E}\lbrace\hat{\boldsymbol{x}}_{\mathrm{s}} \hat{\boldsymbol{x}}_{\mathrm{s}}^{H} \rbrace \!\right)\left(\!{\boldsymbol{I}\odot \mathbb{E}\lbrace \hat{\boldsymbol{x}}_{\mathrm{i}} \hat{\boldsymbol{x}}_{\mathrm{i}}^{H} \rbrace+\boldsymbol{I}\odot\mathbb{E}\lbrace\hat{\boldsymbol{x}}_{\mathrm{n}} \hat{\boldsymbol{x}}_{\mathrm{n}}^{H} \rbrace} \! \right)^{-1} \bigg]_{1,1} \notag \end{array} $$

Before, we derive the different covariance matrices, we recapitulate a required property of circulant matrices. A circulant matrix $\boldsymbol {C} \in \mathbb {C}^{N \times N}$ is fully described by its first column ***c***, as its eigenvectors are the DFT basis-vectors and its eigenvalues are the DFT of $\boldsymbol {c} = (c_{0}, c_{1}, \dots, c_{N-1}) $.

(45) $$\begin{array}{*{20}l} \boldsymbol{C} &=\left(\begin{array}{ccccc} c_{0} & c_{N-1} & \dots & c_{1} \\ c_{1} & c_{0} & & c_{2} \\ \vdots & & \ddots & \vdots \\ c_{N-1} & \dots & c_{1} & c_{0} \\ \end{array}\right) \end{array} $$

(46) $$\begin{array}{*{20}l} &=\boldsymbol{D}^{H} \text{Diag} \left(\boldsymbol{D}\boldsymbol{c} \right)\boldsymbol{D} = \boldsymbol{D}^{H} \boldsymbol{\Lambda} \boldsymbol{D} \end{array} $$

The main diagonal elements *c*_0_ of ***C*** are given by 
(47)

#### $\mathbb {E}\lbrace \hat {\boldsymbol {x}}_{\mathrm {s}} \hat {\boldsymbol {x}}_{\mathrm {s}}^{H} \rbrace $:

The input-output matrix ***K*** is of block-circulant structure, as illustrated in Fig. 3c. The eigenvalues of the diagonal blocks are given by $\text {diag}\left (\boldsymbol {\Lambda }\right)=\boldsymbol {S}^{(l)} \text {diag}\left (\boldsymbol {F} \boldsymbol {H}_{\text {eff}} \right)$ and the diagonal elements of the *l*th diagonal block are then  as asserted by Eq. (47), thus 
(48)

Assuming zero mean, white data with variance, ${\sigma _{x}^{2}}$ the diagonal elements of $\mathbb {E}\lbrace \hat {\boldsymbol {x}}_{\mathrm {s}} \hat {\boldsymbol {x}}_{\mathrm {s}}^{H} \rbrace $ are given by .

#### $\mathbb {E}\lbrace \hat {\boldsymbol {x}}_{\mathrm {i}} \hat {\boldsymbol {x}}_{\mathrm {i}}^{H} \rbrace $:

If ***C*** is circulant

(49) $$ \tilde{\boldsymbol{C}}=\boldsymbol{C}-c_{0} \boldsymbol{I} = \left(\begin{array}{ccccc} 0 & c_{N-1} & \dots & c_{1} \\ c_{1} & 0 & & c_{2} \\ \vdots & & \ddots & \vdots \\ {c_{N-1}} & \dots & c_{1} & 0 \\ \end{array} \right)  $$

is circulant as well and the diagonal elements of $\tilde {\boldsymbol {C}} \tilde {\boldsymbol {C}}^{H}$ are the sum of the magnitude squares of $\tilde {\boldsymbol {c}} = (0, c_{1}, \dots, c_{N-1}) $. Using Parseval’s theorem, we arrive at

(50) $$\begin{array}{@{}rcl@{}}  \sum_{i=1}^{N-1} |c_{i}|^{2} =& \frac{1}{N} \sum_{j=1}^{N-1} |[\boldsymbol{\!\Lambda}]_{j,j}|^{2} \\ =&\frac{1}{N}\sum_{j=0}^{N-1} |[\boldsymbol{\!\Lambda}]_{j,j}|^{2} - \bigg| \frac{1}{N}\sum_{j=0}^{N-1} [\boldsymbol{\!\Lambda}]_{j,j}\bigg|^{2}~. \notag \end{array} $$

The inter-layer interference consists of *L*−1***C***-type blocks, where we simply average the magnitude squares of the eigenvalues, i.e. the corresponding block-part of ***FH***_eff_. The intra-layer interference is described via a $\tilde {\boldsymbol {C}}$ block and is given in Eq. (50). Both contributions can be compactly written as 
(51)

#### $\mathbb {E}\lbrace \hat {\boldsymbol {x}}_{\mathrm {n}} \hat {\boldsymbol {x}}_{\mathrm {n}}^{H} \rbrace $:

The noise covariance matrix is circulant as well and the detailed derivations can be found in [30].

#### SISO MMSE SC-FDMA SINR expression

For a SISO system and a one-tap equalizer, the expression ***FH***_eff_ is of a diagonal shape. [30] has shown, that the MMSE equalizer for SC-FDM equals the OFDM expression, i.e. $\boldsymbol {F}=\left (\frac {{\sigma _{n}^{2}}}{{\sigma _{x}^{2}}} \boldsymbol {I} + \boldsymbol {H}_{\text {eff}}^{H} \boldsymbol {H}_{\text {eff}}\right)^{-1} \boldsymbol {H}_{\text {eff}}^{H}$. Thus, the elements on the main diagonal of ***FH***_eff_ are simply given by $|\boldsymbol {H}_{k}|^{2}\left (\frac {{\sigma _{n}^{2}}}{{\sigma _{x}^{2}}}+|\boldsymbol {H}_{k}|^{2}\right)^{-1}$, and we rewrite (??) to (55), where we have used the identity

(52) $$ \frac{1}{N_{\text{SC}}} \sum\limits_{k=1}^{N_{\text{SC}}} \frac{|\boldsymbol{H}_{k}|^{2}}{\frac{{\sigma_{n}^{2}}}{{\sigma_{x}^{2}}}+|\boldsymbol{H}_{k}|^{2}} =1 - \frac{{\sigma_{n}^{2}}}{{\sigma_{x}^{2}}} \frac{1}{N_{\text{SC}}} \sum\limits_{k=1}^{N_{\text{SC}}} \frac{1}{\frac{{\sigma_{n}^{2}}}{{\sigma_{x}^{2}}}+|\boldsymbol{H}_{k}|^{2}}  $$

from [23].

(53) $$ {\fontsize{7.5}{6} \begin{aligned} &\text{SINR}_{\text{MMSE}}^{\text{SC-FDM}}\\ &\,\,\,= \frac{\frac{{\sigma_{x}^{2}}}{N_{\text{SC}}} \left(\sum\limits_{k=1}^{N_{\text{SC}}} \frac{|\boldsymbol{H}_{k}|^{2}}{\frac{{\sigma_{n}^{2}}}{{\sigma_{x}^{2}}}+|\boldsymbol{H}_{k}|^{2}}\right)^{2}}{{\sigma_{x}^{2}} \sum\limits_{k=1}^{N_{\text{SC}}} \left(\frac{|\boldsymbol{H}_{k}|^{2}}{\frac{{\sigma_{n}^{2}}}{{\sigma_{x}^{2}}}+|\boldsymbol{H}_{k}|^{2}}\right)^{2}-\frac{{\sigma_{x}^{2}}}{N_{\text{SC}}} \left(\sum\limits_{k=1}^{N_{\text{SC}}} \frac{|\boldsymbol{H}_{k}|^{2}}{\frac{{\sigma_{n}^{2}}}{{\sigma_{x}^{2}}}+|\boldsymbol{H}_{k}|^{2}}\right)^{2} +{\sigma_{n}^{2}} \sum\limits_{k=1}^{N_{\text{SC}}} \frac{|\boldsymbol{H}_{k}|^{2}}{\left(\frac{{\sigma_{n}^{2}}}{{\sigma_{x}^{2}}}+|\boldsymbol{H}_{k}|^{2}\right)^{2} }} \end{aligned}}  $$

(54) $$\begin{array}{@{}rcl@{}} &=& \frac{\frac{1}{N_{\text{SC}}} \left(\sum\limits_{k=1}^{N_{\text{SC}}} \frac{|\boldsymbol{H}_{k}|^{2}}{\frac{{\sigma_{n}^{2}}}{{\sigma_{x}^{2}}}+|\boldsymbol{H}_{k}|^{2}}\right)^{2}}{\left(\sum\limits_{k=1}^{N_{\text{SC}}} \frac{|\boldsymbol{H}_{k}|^{2}}{\frac{{\sigma_{n}^{2}}}{{\sigma_{x}^{2}}}+|\boldsymbol{H}_{k}|^{2} }\right) -\frac{1}{N_{\text{SC}}} \left(\sum\limits_{k=1}^{N_{\text{SC}}} \frac{|\boldsymbol{H}_{k}|^{2}}{\frac{{\sigma_{n}^{2}}}{{\sigma_{x}^{2}}}+|\boldsymbol{H}_{k}|^{2}}\right)^{2}}\\ &=& \frac{\frac{1}{N_{\text{SC}}} \sum\limits_{k=1}^{N_{\text{SC}}} \frac{|\boldsymbol{H}_{k}|^{2}}{\frac{{\sigma_{n}^{2}}}{{\sigma_{x}^{2}}}+|\boldsymbol{H}_{k}|^{2}}}{ 1 -\frac{1}{N_{\text{SC}}} \sum\limits_{k=1}^{N_{\text{SC}}} \frac{|\boldsymbol{H}_{k}|^{2}}{\frac{{\sigma_{n}^{2}}}{{\sigma_{x}^{2}}}+|\boldsymbol{H}_{k}|^{2}}} \end{array} $$

(55) $$\begin{array}{@{}rcl@{}} &=&\frac{{\sigma_{x}^{2}}}{{\sigma_{n}^{2}}}\frac{1- \frac{{\sigma_{n}^{2}}}{{\sigma_{x}^{2}}} \frac{1}{{N_{\text{SC}}}} \sum\limits_{k=1}^{N_{\text{SC}}} \frac{1}{\frac{{\sigma_{n}^{2}}}{{\sigma_{x}^{2}}}+|\boldsymbol{H}_{k}|^{2}} }{\frac{1}{{N_{\text{SC}}}}\sum\limits_{k=1}^{N_{\text{SC}}} \frac{1}{\frac{{\sigma_{n}^{2}}}{{\sigma_{x}^{2}}}+|\boldsymbol{H}_{k}|^{2}}}  \end{array} $$
